# Palyno-Morphological Characteristics as a Systematic Approach in the Identification of Halophytic Poaceae Species from a Saline Environment

**DOI:** 10.3390/plants11192618

**Published:** 2022-10-05

**Authors:** Moona Nazish, Ashwaq T. Althobaiti

**Affiliations:** 1 Department of Botany, Rawalpindi Women University, Rawalpindi 46300, Pakistan; 2 Department of Biology, College of Sciences, Taif University, Ta’if 21974, Saudi Arabia

**Keywords:** pollen morphology, grasses, halophytes, Salt Range, micromorphological characteristics, SEM

## Abstract

Pollen morphology of 12 salt-tolerant grasses (*Calamagrostis pseudophragmites*, *Cenchrus biflorus*, *Cenchrus ciliaris*, *Cenchrus pennisetiformis*, *Cymbopogon jwarancusa*, *Dactyloctenium aegyptium*, *Echinochloa colona*, *Echinochloa crus-galli*, *Saccharum griffithii*, *Saccharum ravennae*, *Saccharum spontaneum*, and *Urochloa ramosa*) from the Salt Range was studied using scanning electron microscopy (SEM) and light microscopy (LM). The main aim was the elucidation of taxonomic significance of pollen morphology, which might be useful for taxonomists in the identification of halophytic Poaceae taxa. The described pollen morphology is a good source of taxonomic characteristics that can help in species and genera differentiation. The investigated variations in the pollen micromorphological features and exploration of new palynological traits support and strengthen the systematics of Poaceae. The pollen shape of all studied species was sub-spheroidal, and exine ornamentation includes microechinate-areolate (11 spp.) and microechinate (1 spp.). Two types of pollen apertures were reported, i.e., monoporate (11 spp.) and diporate (*Cenchrus pennisetiformis*). The annulus was found in all species while operculum was observed only in three species. The univariate and multivariate analyses were used to analyze the quantitative data. The highest pollen viability values (%) depicted the adaptability of Poaceae taxa in the salt region. Overall, the pollen characteristics in polar and equatorial view, apertures, annulus, operculum, and surface ornamentation of pollen grains of the family Poaceae are of great taxonomic significance for the species identification.

## 1. Introduction

Poaceae is one of the largest families consisting of 800 genera and 12,000 species distributed worldwide [1]. About 158 genera and 492 species of this family were reported from Pakistan [2]. Poaceae are an economically important family and accounts for global gross primary productivity of about 25% [3]. Poaceae rank as the third largest family, after Orchidaceae and Asteraceae, based on the number of genera, and rank fifth largest, after Fabaceae, Rubiaceae, and Asteraceae, based on the number of species [4]. Its members are more widespread than other angiosperm families. It is a homogenous taxon. Members of the family are mostly herbaceous, subshrubs, shrubs, and culms, morphologically characterized with round stem and leaf with sheath, ligule, and blade with parallel venation. Flowers are arranged in spikelets, and usually contain lemma and palea (2 bracts) with three stamens. Some grasses do not flower in their life cycle for a longer period [5]; although, some Poaceae species develop new inflorescence continuously [6]. Their greater adaptability allows them to survive under varied conditions.

Grasses make up 23% of the vegetation cover of the world [7]. The distribution of grasses in a region depends on the physical and chemical nature of the soil [4]. The perennial cover of grasses plays an important role in the renewal of depleted soils because these are soil stabilizers [8]. The most productive and fertile soils of the world show a vegetation cover of grasses. In any geographic region, plants signify the ecological conditions and become an indicator of soil characteristics because plant and soil characteristics are independent of each other [9]. Kearney et al. (1914) and Hilgard [10,11] reported that some grasses are salinity indicators. The presence of halophytic grasses in saline habitats show the significance of specific characteristics that enable them to adopt several environmental stresses. Salinity results in several adverse effects on such species also caused by osmotic stress [12]. Grasses have greater potential for adaptations in a saline habitat to minimize the detrimental effects of high salinity. These adaptations include a reduction in leaf area, presence of glands and hairs on the surface of the leaf for salt secretion, root lignifications, and sclerenchyma and epidermal succulence [13,14]. Poaceae represents the second highest number of halophytes (68) out of the total number of halophytes (410) reported from Pakistan [15].

Techniques such as light microscopy (LM) and scanning electron microscopy (SEM) play an important role in pollen characterization and identification of taxa at the species and genus levels [16]. The high magnification of SEM with ultrastructure detail gives information about the qualitative variation, which is not possible to observe using an optical microscope [17]. Many studies were reported in the literature on the palyno-morphological characteristics of Poaceae using SEM and LM techniques and have proven useful for taxonomic classification [18,19,20,21,22]. Some authors in previous studies reported pollen morphometric differences using LM [23,24,25]. Similarly, palynologists distinguished the taxonomic significance of pollen grains using SEM by revealing diversity in the patterns of exine ornamentation [26,27].

The palynological investigations are useful for correct identification and differentiation of various plant taxa and may provide additional information about closely related species and genera [28,29]. Palyno-morphological characteristics are also important in resolving taxonomic problems in grasses [30]. The morphological study of pollen grains may provide important information about the evolutionary history of Poaceae taxa. The pollen features play a significant role at the tribe, genus, and species levels in delimiting Poaceae taxa [31].

Grasses possess great diversity in pollen morphology. Generally, pollen grains in grasses are monoporate, while some species are diporate [32]. Poaceae is in the stenopalynous family with monad-type pollen grains and spheroidal shape, but some species are prolate to oblate spheroidal, small pore, operculate or non-operculate, annulate or non-annulate, and tectum scabrate areolate [2,33,34]. It is always difficult to distinguished pollen grains of closely related genera and species using LM [35]. Similarly, the stenopalynous nature of this family makes it more difficult to differentiate the pollen grains in various taxa of Poaceae. Schüler and Behling [1] studied Poaceae pollen grains to assess the dynamics of palaeoecological grassland in South America and concluded tropical grasses show little variation in size and composition of Poaceae taxa and this is based on the mean size of different parameters of grain and abundance. The increase in abundance of Poaceae pollen is linked with the increased aridity of the region [36].

Pollen pore size and annulus can also be used as a tool to differentiate the closely related species in Poaceae [37]. The pollen morphology in Poaceae taxa showed variation in grain size at the species level [38]. The spheroidal pollen shape with a single pore is utilized by taxonomists to propose the uniform pollen morphology among taxa [39,40]. The genera, such as *Saccharum* and *Cymbopogon*, are problematic taxa as they cause a nuisance in identification for taxonomist because these are difficult to differentiate using morphological markers [41]. Chaturvedi and Datta [42] also reported *Saccharum* with stenopalynous pollen grains. Radaeski et al. (2014) [43] described the pollen characteristics of six taxa of the family of Poaceae with monoporate and spherical shape pollen grains. The palyno-morphological characteristics of *Paspalum plicatulum* and *Paspalum notatum* were also described [44]. Jan [23] reported the same variations in grain size of wild grasses as compared to cultivated taxa of Poaceae. Ahmad et al. (2011) [31] studied five species belonging to the tribe Chlorideae using LM and SEM. They reported oblate spheroidal and prolate spheroidal shape pollen grains with verrucate and rugulate sculpturing. In another study, the pollen morphological characteristics of 45 kinds of grasses from the Azores, Portugal, was conducted [45] and monoporate pollen grains with a sub-circular, circular, and ovalate polar view were observed. Radaeski et al. (2016) [46] studied the pollen grains of 68 species in Poaceae to distinguish grasslands from forests. Their analysis identified large size pollen grains in forest species, medium size pollen in herbaceous species, and small size pollen in grassland species. Dwari and Mondal [47] conducted a study on *Sporobolus diandrus* and reported monad-type, monoaperturate, and prolate-spheroidal shape pollen grains.

The Salt Range of Northern Punjab, Pakistan has a rich diversity of flora, generally, and salt-tolerant grasses in particular [48]. The Poaceae is one of the dominant families in the Salt Range representing diverse halophytes. In the literature, some researchers in Pakistan studied the pollen morphological characteristics in Poaceae generally, while, to the best of our knowledge, no taxonomist and palynologist have studied the salt-tolerant grasses from this region. The use of LM and SEM techniques concerning pollen morphology of Poaceae with particular emphasis on halophytic grasses have been studied for application as an aid in correct identification. It is the first report on pollen morphological investigation of salt-tolerant grasses from North Punjab, Pakistan. The main aim was to evaluate the palyno-morphological characteristics and their pollen viability using LM and SEM techniques as an aid for correction and identification with taxonomic significance.

## 2. Results

**Figure 1:**
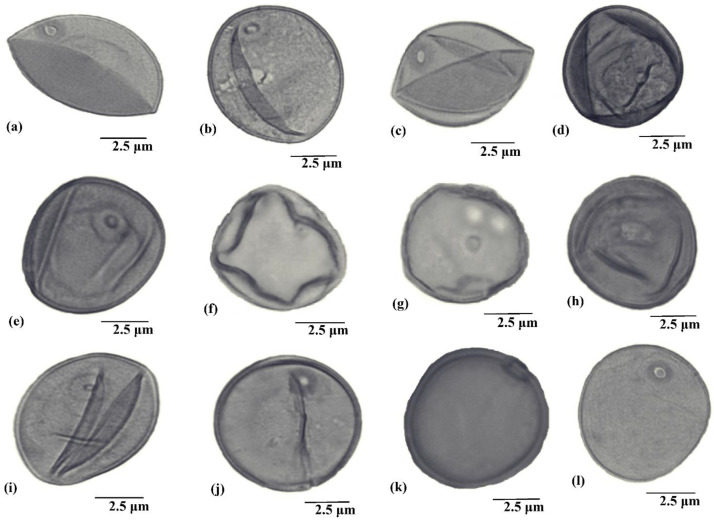
Light microscope pollen micrographs of salt tolerant grasses. (**a**) *Calamagrostis pseudophragmites*, (**b**) *Cenchrus biflorus*, (**c**) *Cenchrus ciliaris*, (**d**) *Cenchrus pennisetiformis*, (**e**) *Cymbopogon jwarancusa*, (**f**) *Dactyloctenium aegyptium*, (**g**) *Echinochloa colona*, (**h**) *Echinochloa crus-galli*, (**i**) *Saccharum griffithii*, (**j**) *Saccharum ravennae*, (**k**) *Saccharum spontaneum*, (**l**) *Urochloa ramosa*.

**Figure 2:**
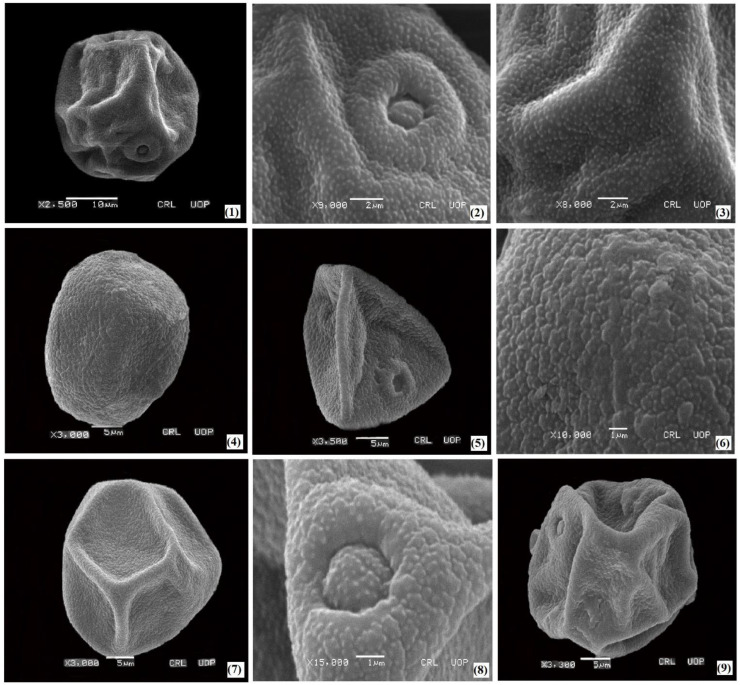
SEM pollen micrographs of salt tolerant grasses. *Urochloa ramosa* (**1**–**3**), *Calamagrostis pseudophragmites* (**4**–**6**), *Cenchrus biflorus* (**7**–**9**).

**Figure 3:**
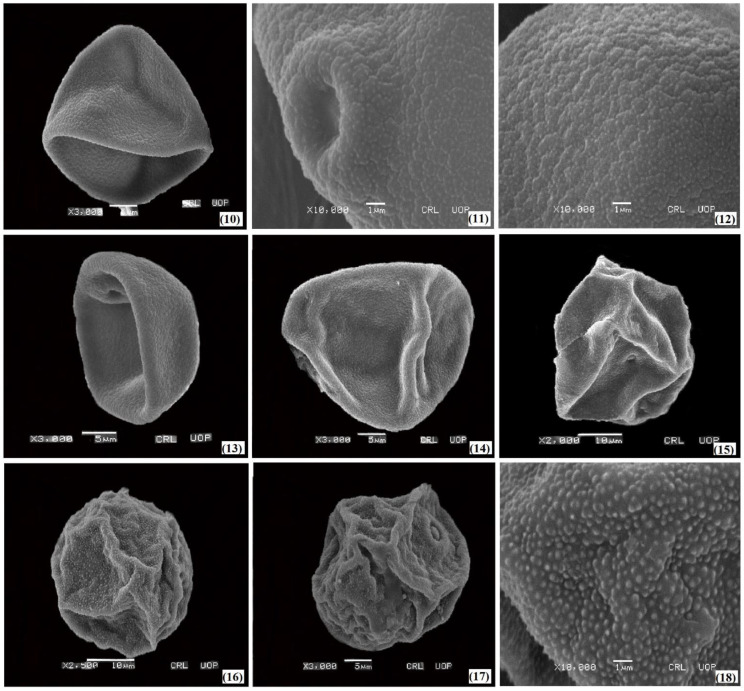
SEM pollen micrographs of salt tolerant grasses. *Cenchrus ciliaris* (**10**–**13**), *Cenchrus pennisetiformis* (**14**,**15**), *Cymbopogon jwarancusa* (**16**–**18**).

**Figure 4:**
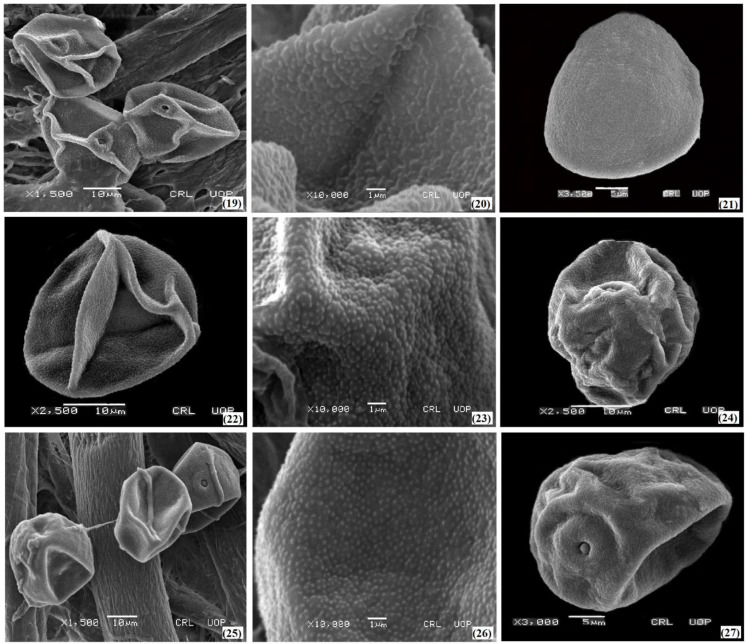
SEM pollen micrographs of salt tolerant grasses. *Dactyloctenium aegyptium* (**19**–**21**), *Echinochloa colona* (**22**–**24**), *Echinochloa crus-galli* (**25**–**27**).

**Figure 5:**
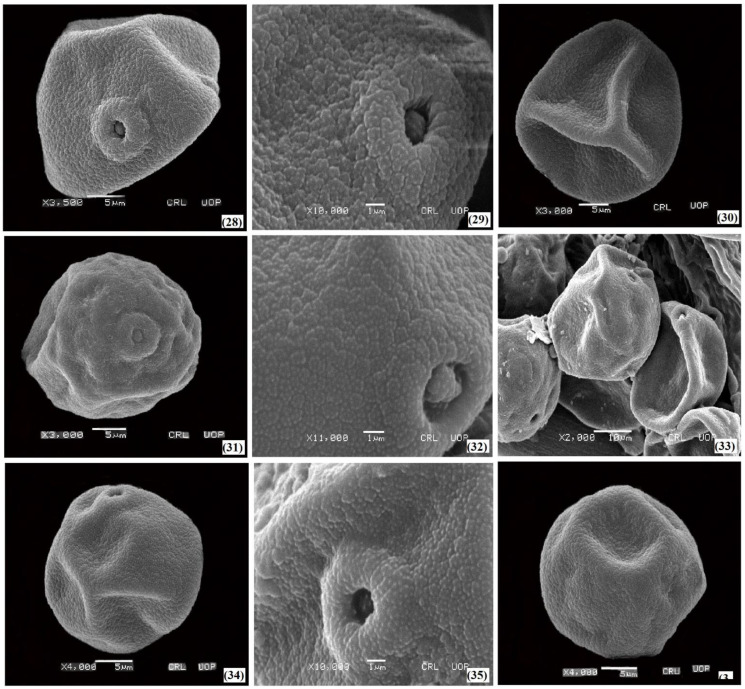
SEM pollen micrographs of salt tolerant grasses. *Saccharum griffithii* (**28**–**30**), *Saccharum ravennae* (**31**–**33**), *Saccharum spontaneum* (**34**–**36**).

The palyno-morphological characteristics of 12 halophytic grasses belonging to seven genera were investigated using LM and SEM. The quantitative and qualitative morphological pollen characteristics of salt-tolerant grasses are presented in Table 1. The pollen micrographs were taken using LM and SEM and illustrated in Figure 1, Figure 2, Figure 3, Figure 4 and Figure 5. The micromorphological pollen features of halophytic grasses showed variation in size and exine sculpturing, which can be used as potential taxonomic characteristics as an aid for correct identification.

### 2.1. Diversity in Pollen Size and Shape

Medium sized and sub-spheroidal shaped pollen grains were observed in all studied halophytic grasses. The pollen grains of salt-tolerant grasses exhibited uniformity in pollen shape, i.e., sub-spheroidal. The polar to equatorial diameter ranges from 38.55 µm and 44.55 µm to 32.75 µm and 34.0 µm among studied *Cenchrus* species. The *P*/*E* ratio ranges from 0.86 (*Cenchrus pennisetiformis*) to 1.05 (*Cenchrus biflorus*). *Saccharum griffithii* show the largest polar diameter (31.75 µm) and equatorial diameter (33.85 µm). The highest *P*/*E* ratio was calculated for *Saccharum spontaneum* (1.10).

Similarly, the palyno-morphological characteristics of two species of genus *Echinochloa* were reported with polar and equatorial diameters varying from 27.35 µm and 30.05 µm (*Echinochloa crus-galli*) to 29.65 µm and 30.35 µm (*Echinochloa colona*). *Urochloa* and *Cymbopogon* were represented with a single species each. There were considerable variations observed in the *P*/*E* ratio of *Urochloa ramosa* (0.94) and *Cymbopogon jwarancusa* (0.99). The average equatorial and polar diameters (29.60–27.85 µm) were reported in *Urochloa ramosa* (31.50 µm), and 31.20 µm in *Cymbopogon jwarancusa*.

The highly salt-tolerant grass species belongs to the genera *Calamagrostis* and *Dactyloctenium*. The same polar diameter (27.85 µm) was reported in *Calamagrostis pseudophragmites* and *Dactyloctenium aegyptium*. The *P*/*E* ratios of *Calamagrostis pseudophragmites* and *Dactyloctenium aegyptium* were 1.03 and 0.97, respectively. The lack of statistical differences between studied taxa for many variables also demonstrates the homogeneous nature of this family.

### 2.2. Pollen Aperture

Aperture is one of the important features in pollen identification of closely related taxa [49]. The pollen apertures are reported, from monoporate to diporate, in the three studied species of the genus *Cenchrus*. All of the three species, *Cenchrus biflorus*, *Cenchrus ciliaris*, and *Cenchrus pennisetiformis*, have visible annulus. In *Cenchrus pennisetiformis*, 10 diporate grains were observed as a proportion of the total number of 25 grains studied. The pore length and width ranges from 1.76 µm to 1.66 µm in *Cenchrus pennisetiformis* to 1.99 µm to 1.82 µm in *Cenchrus ciliaris*. Monoporate pollen grains without operculum was observed in three species of the genus *Saccharum*. The species *Saccharum griffithii*, *Saccharum ravennae*, and *Saccharum spontaneum* have clear annulus. The pore width and length vary from *Saccharum griffithii* (1.96 µm and 1.94 µm) to *Saccharum spontaneum* (1.76 µm and 1.78 µm). Similarly, monoporate pollen grains were reported in *Echinochloa colona* and *Echinochloa crus-galli*. A large size aperture was reported in *Echinochloa colona* with a length of 1.96 µm and width of 1.86 µm. The aperture with annulus and opercula was observed in *Echinochloa colona*, while no opercula have been observed in *Echinochloa crus-galli*. Pollen grains of *Cymbopogon jwarancusa* with visible annulus and operculum was also observed (Figure 3). The difference in the pore length of *Urochloa ramosa* (1.90 µm) and *Cymbopogon jwarancusa* (1.80 µm) has been noticed. Similarly, in *Calamagrostis pseudophragmites* and *Dactyloctenium aegyptium* monoporate pollen grains were reported with a slight variation in aperture size.

### 2.3. Exine Ornamentation

Regarding the exine ornamentation, a similar variation was observed in such halophytic species. The exine ornamentation in three species of the genus *Cenchrus* with microechinate-areolate pollen was presented (Figure 2 and Figure 3). Exine thickness varies among the *Saccharum* spp, from 0.84 µm (*Saccharum griffithii*) to 0.92 µm (*Saccharum ravennae*). Microechinate-areolate exine sculpturing was seen in *Saccharum spontaneum*, *Saccharum griffithii*, and *Saccharum ravennae* (Figure 5). In both *Echinochloa* species, microechinate-areolate exine ornamentation was observed with maximum exine thickness (1.04 µm) in *Echinochloa colona* (Figure 4). Among the other examined salt-tolerant grasses, the maximum exine thickness was found in *Dactyloctenium aegyptium* (1.06 µm), while the minimum was found in *Cymbopogon jwarancusa* (0.64 µm) with microechinate exine ornamentation.

### 2.4. Pollen Viability

The viability of pollen grains is an important characteristic which provides information about the pollen incompatibility and fertility. The pollen viability plays a significant role in plants’ diversity and their distribution in various habitats. In this study, the highest and lowest pollen viability percentages confirm their stability in the Salt Range of Northern Punjab, Pakistan. The maximum pollen viability (91.58%) and minimum pollen non-viability (8.41%) was observed in *Saccharum spontaneum* and *Dactyloctenium aegyptium,* while the maximum pollen non-viability (21.12% and 19.85%) and minimum pollen viability (78.87% and 80.14%) was found in *Echinochloa crus-galli* and *Cenchrus pennisetiformis* among the studied salt-tolerant grasses (Table 2).

### 2.5. Principal Component Analysis

**Figure 6:**
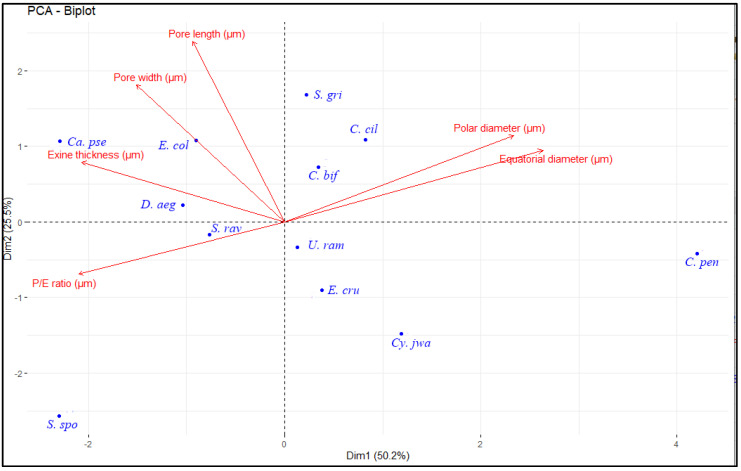
Principal component analysis (PCA) among the studied salt-tolerant Poaceae species based on palyno-morphological characteristics. (*Calamagrostis pseudophragmites*, *Cenchrus biflorus*, *Cenchrus ciliaris*, *Cenchrus pennisetiformis*, *Cymbopogon jwarancusa*, *Dactyloctenium aegyptium*, *Echinochloa colona*, *Echinochloa crus-galli*, *Saccharum griffithii*, *Saccharum ravennae*, *Saccharum spontaneum*, and *Urochloa ramosa*).

Multivariate statistical techniques, such as PCA, facilitated the testing of potential correlations between the quantitative pollen features (Figure 6). The first and second axes from the PCA described 75.7% of the variance. The first axis separates grasses that have bigger polar and equatorial diameters, thinness exine, and small *P*/*E* ratios, such as *Cenchrus pennisitiformis*, from those with the smallest polar and equatorial diameters and thickest exine on the negative side. The first principal component described 50.2% of the variance, with the polar diameter and equatorial diameter being the most significant variables. The second principal component explained 25.5% of the variance in the data, with pore length and pore width being the most significant variables (Table 3).

## 3. Discussion

The findings of this study provide the palyno-morphological characteristics of 12 salt-tolerant grasses belonging to seven genera using LM and SEM distributed in the Salt Range of Northern Punjab, Pakistan. Palynology is one of the major disciplines used by the modern taxonomist for the identification and differentiation of closely related taxa [50]. Pollen micromorphology acts as an additional and significant tool for taxonomic description and implication [51]. Mbagwu et al. (2009) [52] studied palynological attributes to solve the taxonomic problem of closely related taxa. The analysis of pollen grains using LM and SEM aids in solving taxonomic problems related to the systematics of grasses [22,30,53]. The morphological dissimilarities and similarities play an important role in the identification of plant species. In this study, the pollen size, shape, polar diameter, equatorial diameter, *P*/*E* ratio, exine ornamentation, apertures, length and width of the pore, and pollen viability and non-viability percentage were studied for their application as an aid in the systematics of grasses.

The Salt Range of Northern Punjab has a rich diversity of grasses [4]. The studied salt-tolerant grasses have pollen grains with monoporate and diporate apertures with or without opercula and annulus and have subspheroidal shape. Perveen and Qaiser [2] reported monoporate and rarely diporate pollen grains in Poaceae. Dórea and Corrêa and Fonseca [19,54] described the Poaceae as a stenopalynous family with little variation in pollen morphological characteristics at the species and genus level.

Exine ornamentation is an important feature from an evolutionary and phylogenetic point of view [55]. The microechinate-areolate and microechinate sculpturing of exine have been observed in all examined halophytic grasses. Siddiqui and Qaiser [56] also reported areolate and scabrate exine sculpturing in Poaceae. Mander et al. (2013) [57] have observed scabrate exine sculpturing in grasses and classified grass pollen grains through quantitative morphometric analysis of exine texture and ornamentation. The study of the exine surface of Poaceae pollen grains under high SEM magnifications displays variations among species [19]. A number of previous studies on the exine ornamentation of Poaceae revealed variation among species and genera [58,59,60]. The quantitative measurements of ornamentation and shape depend on a visual inspection to classify the phenotypic differences and have a wide range of implementations in biological sciences [57].

The position and number of the aperture are of great importance in palynology [61]. The majority of the studied species have monoporate apertures, except for *Cenchrus pennisetiformis* with diporate and monoporate apertures. In the studied species, annulus was present. *Cymbopogon jwarancusa*, *Dactyloctenium aegyptium*, and *Echinochloa colona* were found to have opercula, which was absent in the rest of the species. Morgado et al. (2015) [45] also reported monoporate pollen grains with operculum and annulus in Poaceae. Radaeski et al. (2016) [46] studied monoporate apertures in Poaceae. Pollen grains in Poaceae may provide a strong correlation among size, pore, and annulus [24,62]. The aperture is reported as an important feature used for the identification of closely related taxa [63].

The pollen size and exine sculpturing have greater importance in the grass taxonomy [64]. The pollen size and ornamentation play an important role in taxonomic studies for the correct identification and delimitation [65]. Three species of *Cenchrus* and *Saccharum* were classified on the basis of pollen shape, apertures, and exine sculpturing. Hameed et al. (2008) [66] confirmed that the *Cenchrus ciliaris* is well adapted to the highly saline arid habitat. All studied halophytic grasses have medium sized pollen grains. Our findings are dissimilar from previous research which found variations in pollen grain size of Poaceae [45,67]. The examined species also have variations in exine thickness. The minimum exine thickness was found in *Cymbopogon jwarancusa* (0.64 µm) while the maximum in *Calamagrostis pseudophragmites* (1.1 µm).

Pollen shape is a striking feature used in the delimitation of closely related taxa [68]. All studied genera that are *Calamagrostis*, *Cenchrus, Cymbopogon, Dactyloctenium, Echinochloa, Saccharum*, and *Urochloa* were reported to have a sub-spheroidal shape. The diversity in pollen morphological characteristics using LM and SEM provides evidence regarding variations in pollen type, apertures, and exine ornamentation. The pollen fertility and morphology have closely related functions because various features in pollen grains enable them to survive in a particular geography [69]. Pollen viability and non-viability is a valuable tool to determine the degree of stability of plant species growing in favorable or unfavorable conditions. Pollen fertility is useful for studying genetic variations of the flora [70]. Awan et al. (2001) [71] also indicated the relation of pollen adaptability in an environment with pollen fertility and reported the existence of the ploidy level in correlation with the high pollen fertility level. The PCA analysis revealed that salt-tolerant Poaceae species show little variations in some characteristics, such as among the *P*/*E* ratio, polar and equatorial diameter, and exine thickness. PCA analysis exhibits the maximum contribution at each axis for the total variability [72]. PCA is a useful analysis to investigate the variation pattern and to delimit the species in the genus [73].

SEM and LM techniques have a significant role in plant systematics [74]. This study could be important taxonomically for the correct identification and delimitation of Poaceae taxa. The inter-species relationship exhibited by the palyno-morphological characteristics suggests the basis for salt-tolerant grasses to be in the same family. The role of palynology in plant taxonomy has proved valuable in solving critical and disputed taxonomic problems.

## 4. Materials and Methods

### 4.1. Collection and Identification

**Figure 7:**
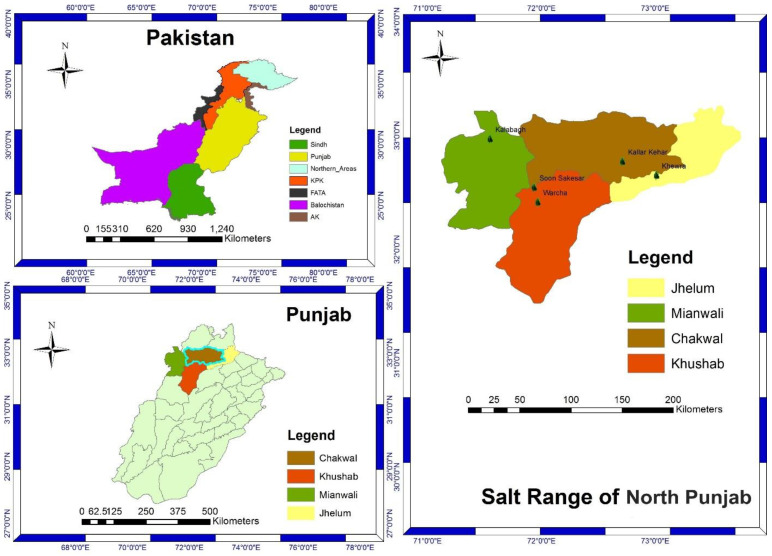
Map of the study area.

A total of 12 salt-tolerant grasses were collected during extensive field trips through a planned schedule from the Salt Ranges in Northern Punjab including Kalabagh, Kallar Kahar, Khewra, Soon Sakesar, and Warcha (Figure 7). These areas are considered to be rich with halophytic grasses. About five specimens of each species were collected randomly in different seasons of the year from February 2017 to March 2019. The flowering seasons with altitude and locality of salt-tolerant grasses are given in Table 4. The species were identified with the help of the available literature (http://www.efloras.org) (accessed on 7 June 2017) and the Flora of Pakistan. The identified species were further reconfirmed by comparing them with the herbarium specimens in the Herbarium of Pakistan (ISL). The botanical names were authenticated and validated with the help of Tropicos (Missouri Botanical Gardens) (http://www.tropicos.org/) (accessed on 25 July 2017). The collected grasses were pressed, dried, and preserved using standard herbarium techniques and mounted on herbarium sheets with voucher accession numbers, and then deposited in the Herbarium of Pakistan, Department of Plant Sciences, Quaid-i-Azam University, Islamabad, Pakistan for future reference.

### 4.2. Light Microscopic Analysis of Pollen in Salt-Tolerant Grasses

Pollen samples were processed for LM using the method of acetolysis [75]. The pollen grains were stained following the procedure of [76]. To obtain the mean size of pollen and variation, 25 readings were counted for the polar and equatorial diameter, exine thickness, and pore size [77]. To see the variations, 5 pollen grains from 5 collected specimens of each taxon were included in this analysis. The shape of pollen grains was determined based on the polar to equatorial diameter (*P*/*E*) ratio. The classification of [78] was followed to describe the pollen types. A biological microscope (Model: MX5300H, Meiji Techno, Iruma-gun Saitama, Japan) was used for the light microscopic study of the pollen grains. A magnification of 40× was used for measurements. The Leica dialux Light microscope (Model 1000, Mannheim, Germany) was used for pollen micrographs at different resolutions. In order to describe the pollen qualitatively, the terminologies reported by [79] were followed.

### 4.3. Scanning Electron Microscopic Analysis of Pollen in Salt-Tolerant Grasses

In this study, SEM was used to differentiate closely related taxa of Poaceae using various characteristics presented by electron microphotographs. Pollen grains of salt-tolerant grasses were prepared for SEM using the techniques of [80]. The pollen grains were placed on the metallic stub. The pollen grains were coated with gold palladium (2.3 nm) and examined under SEM (Model JEOL JSM-5910, Peabody, USA). Polaroid P/N 665 film was used to take pollen microphotographs.

### 4.4. Statistical Analysis

The results were analyzed quantitatively using SPSS (16.0) software, IBM (University of Stanford) to calculate the arithmetic mean and standard error for all quantitative characteristics of pollen grains. The pollen grains were quantitatively described by measuring the polar diameter, equatorial diameter, pore length, pore width, and exine thickness. The pollen viability assessment is crucial to ensure fertility, to evaluate the germination rate of pollen grains under certain conditions, and to evaluate pollen grains dispersal rate. Non-viable pollen grains refers to their inability to grow, live, and germinate [81]. A total of 25 slides of pollen grains were prepared for the light microscopic study to determine the pollen viability. The pollens were placed on a glass slide and crushed using a glass rod after pouring 1–2 drops of acetic acid. The debris was removed from the glass slide with the help of a needle and stained with glycerin jelly. The pink colored pollen grains were viable, while the brown colored or non-stained pollen grains were non-viable. The viability of pollen grains can vary due to taxonomic reasons, length of time since collection, time of year of collection, or other factors. The pollen viability and non-viability percentages were determined using the following formula

$$Pollen\;viability=\frac{F}{F+S}\times 100$$

$$Pollen\;non-viability=\frac{S}{S+F}\times 100$$

where, *F* is the number of fertile pollen grains and *S* is the number of sterile pollen grains.

The principal component analysis (PCA) was performed using the software XLSTAT to discriminate between the quantitative pollen morphological characteristics. The used metric variables were polar and equatorial diameter, pore size, *P*/*E* ratio, and exine thickness.

The *P*/*E* ratio of each salt-tolerant grass was calculated using the following formula

$$P/E=\frac{P}{E}\times 100$$

where, *E* is the equatorial diameter and *P* is the polar diameter of the same pollen.

## 5. Conclusions

Pollen micromorphological characteristics are a valuable taxonomic tool in plant systematics. This study elucidates the significance of the LM and SEM techniques in the identification of salt-tolerant species of the family Poaceae. The findings revealed the similarities as well as dissimilarities in pollen micromorphological features. The basic palyno-morphological characteristics are the same, but examined individual differences in halophytic grasses provide evidence which can be used as tool for correct species identification. This is the first reported study on the palyno-morphological investigation of halophytic grasses from the Salt Range of Northern Punjab. The micromorphological features and viability of pollen grains of halophytic grasses may contribute to the taxonomic position of Poaceae, and will be useful for the correct identification of problematic taxa in the future.

## Figures and Tables

**Table 1:** Qualitative and quantitative pollen characteristics of salt-tolerant grasses from Northern Punjab, Pakistan.

Plant Name	Pollen Size	Pollen Shape	Annulus	Operculum	Aperturate	Polar Diameter (µm) Min (Mean ± Std Error) Max	Equatorial Diameter (µm) Min (Mean ± Std Error) Max	P/E Ratio (µm)	Exine Thickness (µm) Min (Mean ± Std Error) Max	Pore Length (µm) Min (Mean ± Std Error) Max	Pore Width (µm) Min (Mean ± Std Error) Max	Sculpturing (SEM)
*Calamagrostis* *pseudophragmites* (Haller f.) Koeler	Medium	Subspheroidal	+	−	Monoporate	27.25 (27.85 ± 0.48) 29.75	25.50 (26.80 ± 0.54) 28.0	1.03	1.0 (1.1 ± 0.03) 1.2	1.7 (1.94 ± 0.09) 2.2	1.8 (1.96 ± 0.05) 2.1	Microechinate-areolate
*Cenchrus biflorus* Roxb.	Medium	Subspheroidal	+	−	Monoporate	30.50 (35.5 ± 1.66) 40.25	28.0 (33.5 ± 1.60) 37.75	1.05	0.6 (0.72 ± 0.05) 0.9	1.6 (1.86 ± 0.08) 2.1	1.7 (1.96 ± 0.08) 2.2	Microechinate-areolate
*Cenchrus ciliaris* L.	Medium	Subspheroidal	+	−	Monoporate	27.25 (32.75 ± 2.19) 38.0	30.25 (34.05 ± 1.83) 40.25	0.96	0.5 (0.72 ± 0.10) 0.9	1.9 (1.99 ± 0.08) 2.3	1.8 (1.82 ± 0.06) 2.1	Microechinate-areolate
*Cenchrus pennisetiformis* Hochst. and Steud.	Medium	Subspheroidal	+	−	Diporate, Monoporate	32.50 (38.55 ± 1.58) 41.75	43.0 (44.55 ± 0.91) 47.75	0.86	0.6 (0.72 ± 0.05) 1.1	1.6 (1.76 ± 0.05) 1.9	1.5 (1.66 ± 0.08) 1.9	Microechinate-areolate
*Cymbopogon jwarancusa* (Jones) Schult.	Medium	Subspheroidal	+	+	Monoporate	25.50 (31.20 ± 2.19) 38.25	29.75 (31.50 ± 0.77) 34.0	0.99	0.5 (0.64 ± 0.07) 0.9	1.6 (1.80 ± 0.07) 2.0	1.5 (1.72 ± 0.06) 1.9	Microechinate
*Dactyloctenium aegyptium* (L.) Willd.	Medium	Subspheroidal	+	+	Monoporate	23.0 (27.85 ± 1.48) 32.25	26.25 (28.65 ± 0.69) 30.25	0.97	0.9 (1.06 ± 0.05) 1.2	1.8 (1.92 ± 0.03) 2.0	1.7 (1.80 ± 0.03) 1.9	Microechinate-areolate
*Echinochloa colona* (L.) Link	Medium	Subspheroidal	+	+	Monoporate	25.50 (29.65 ± 1.84) 35.75	28.0 (30.35 ± 0.88) 33.25	0.97	0.9 (1.04 ± 0.06) 1.2	1.5 (1.96 ± 0.10) 2.1	1.6 (1.86 ± 0.07) 2.0	Microechinate-areolate
*Echinochloa crus-galli* (L.) P. Beauv.	Medium	Subspheroidal	+	−	Monoporate	22.75 (27.35 ± 1.65) 32.75	24.50 (30.05 ± 1.77) 33.50	0.91	0.6 (0.78 ± 0.09) 1.1	1.8 (1.78 ± 0.08) 2.2	1.7 (1.84 ± 0.03) 1.9	Microechinate-areolate
*Saccharum griffithii* Munro ex **Boiss.**	Medium	Subspheroidal	+	−	Monoporate	26.25 (31.75 ± 1.97) 35.50	31.25 (33.85 ± 1.01) 37.25	0.93	0.5 (0.84 ± 0.12) 1.1	1.6 (1.96 ± 0.11) 2.3	1.8 (1.94 ± 0.05) 2.1	Microechinate-areolate
*Saccharum ravennae* (L.) L.	Medium	Subspheroidal	+	−	Monoporate	24.75 (29.80 ± 2.48) 38.5	23.0 (28.70 ± 2.57) 38.0	1.03	0.6 (0.92 ± 0.10) 1.2	1.7 (1.90 ± 0.08) 2.1	1.6 (1.80 ± 0.07) 2.0	Microechinate-areolate
*Saccharum spontaneum* L.	Medium	Subspheroidal	+	−	Monoporate	20.25 (23.10 ± 1.04) 25.5	15.25 (20.95 ± 1.94) 25.25	1.10	0.7 (0.9 ± 0.08) 1.1	1.6 (1.76 ± 0.05) 1.9	1.7 (1.78 ± 0.03) 1.9	Microechinate-areolate
*Urochloa ramosa* (L.) T.Q. Nguyen	Medium	Subspheroidal	+	−	Monoporate	22.25 (27.85 ± 2.88) 32.25	25.50 (29.60 ± 1.65) 33.75	0.94	0.6 (0.78 ± 0.05) 0.9	1.7 (1.90 ± 0.07) 2.1	1.6 (1.78 ± 0.07) 2.0	Microechinate-areolate

**Table 2:** Pollen viability and non-viability percentages for salt-tolerant grasses.

Botanical Name	No. of Viable Pollen	No. of Non-Viable Pollen	Viability (%)	Non-Viability (%)
*Calamagrostis pseudophragmites* (Haller f.) Koeler	95	16	85.58	14.41
*Cenchrus biflorus* Roxb.	104	23	81.88	18.11
*Cenchrus ciliaris* L.	93	12	88.57	11.42
*Cenchrus pennisetiformis* Hochst. and Steud.	113	28	80.14	19.85
*Cymbopogon jwarancusa* (Jones) Schult.	67	8	89.33	10.66
*Dactyloctenium aegyptium* (L.) Willd.	69	7	90.78	9.21
*Echinochloa colona* (L.) Link	113	25	81.88	18.11
*Echinochloa crus-galli* (L.) P. Beauv.	56	15	78.87	21.12
*Saccharum griffithii* Munro ex Boiss.	102	17	85.71	14.28
*Saccharum ravennae* (L.) L.	62	11	84.93	15.06
*Saccharum spontaneum* L.	98	9	91.58	8.41
*Urochloa ramosa* (L.) T.Q. Nguyen	87	19	82.07	17.92

**Table 3:** Correlation coefficients for metric variables of principal component analysis (PCA) using palynological characteristics of salt-tolerant grasses.

Variables	PC1	PC2	PC3	PC4	PC5	PC6
Polar diameter (µm)	0.4744565	0.3249055	−0.32621870	0.43538549	−0.17548822	−0.58527955
Equatorial diameter (µm)	0.5349302	0.2709428	0.01092927	0.25624982	0.03831308	0.75709210
P/E ratio (µm)	−0.4265074	−0.1965046	−0.60566071	0.35522178	−0.45418376	0.28317327
Exine thickness (µm)	−0.4201325	0.2252430	0.53173029	0.69077481	0.10909227	−0.03076033
Pore length (µm)	−0.1900636	0.6811253	0.15652833	−0.36410765	−0.58410765	0.04107229
Pore width (µm)	−0.3069148	0.5174421	−0.46840025	−0.09406787	0.63903852	0.03793646
Eigenvalue	3.011799883	1.532333989	0.758921938	0.360648574	0.333815144	0.002480472
Variation (%)	50.1966647	25.5388998	12.6486990	6.0108096	5.5635857	0.0413412
Cumulative variance (%)	50.19666	75.73556	88.38426	94.39507	99.95866	100.00000

**Table 4:** Taxon Sampling and deposition in the herbarium.

Taxon	Voucher Specimen Number	Collectors	Altitudes (m)	Flowering Period	Locality	District
*Calamagrostispseudophragmites* (Haller f.) Koeler	ISL.129931	Moona Nazish and Farhat Ullah	181.00	May–October	Kalabagh	Mianwali
*Cenchrus biflorus* Roxb.	ISL.129955	Moona Nazish	181.03	July–September	Khewra	Jhelum
*Cenchrus ciliaris* L.	ISL.129944	Moona Nazish	152.90	March–October	Khewra	Jhelum
*Cenchrus pennisetiformis* Hochst. and Steud.	ISL.129959	Moona Nazish and Farhat Ullah	153.00	April–August	Sakesar	Khushab
*Cymbopogon jwarancusa* (Jones) Schult.	ISL.129971	Moona Nazish	152.90	June–September	Soon Valley	Khushab
*Dactyloctenium aegyptium* (L.) Willd.	ISL.129953	Moona Nazish	182.08	April–October	Kalabagh	Mianwali
*Echinochloa colona* (L.) Link	ISL.129980	Moona Nazish	169.00	March–November	Warcha	Khushab
*Echinochloa crus-galli* (L.) P. Beauv.	ISL.129970	Moona Nazish	151.70	June–September	Soon Valley	Khushab
*Saccharum griffithii* Munro ex Boiss.	ISL.129961	Moona Nazish	643.00	March–August	Kallar Kahar	Chakwal
*Saccharum ravennae* (L.) L.	ISL.129937	Moona Nazish and Farhat Ullah	181.10	April–September	Khewra	Jhelum
*Saccharum spontaneum* L.	ISL.129981	Moona Nazish	153.01	March–September	Sakesar	Khushab
*Urochloa ramosa* (L.) T.Q. Nguyen	ISL.129973	Moona Nazish	169.01	July–October	Warcha	Khushab

## Data Availability

Not applicable.
